# Radiolabeled Gold Nanoparticles for Imaging and Therapy of Cancer

**DOI:** 10.3390/ma14010004

**Published:** 2020-12-22

**Authors:** Francisco Silva, Maria Paula Cabral Campello, António Paulo

**Affiliations:** 1 CTN—Centro de Ciências e Tecnologias Nucleares, Instituto Superior Técnico, Universidade de Lisboa, Estrada Nacional 10 (km 139,7), 2695-066 Bobadela, Portugal; fsilva@ctn.tecnico.ulisboa.pt (F.S.); pcampelo@ctn.tecnico.ulisboa.pt (M.P.C.C.); 2 DECN—Departamento de Engenharia e Ciências Nucleares, Instituto Superior Técnico, Universidade de Lisboa, Estrada Nacional 10 (km 139,7), 2695-066 Bobadela, Portugal

**Keywords:** gold nanoparticles (AuNPs), nuclear imaging, radionuclide therapy, nanomedicine, nanotechnology

## Abstract

In the Last decades, nanotechnology has provided novel and alternative methodologies and tools in the field of medical oncology, in order to tackle the issues regarding the control and treatment of cancer in modern society. In particular, the use of gold nanoparticles (AuNPs) in radiopharmaceutical development has provided various nanometric platforms for the delivery of medically relevant radioisotopes for SPECT/PET diagnosis and/or radionuclide therapy. In this review, we intend to provide insight on the methodologies used to obtain and characterize radiolabeled AuNPs while reporting relevant examples of AuNPs developed during the last decade for applications in nuclear imaging and/or radionuclide therapy, and highlighting the most significant preclinical studies and results.

## 1. Introduction

### 1.1. General Considerations

During the last decades, progresses in cancer research has been remarkable and cancer survival has steadily improved along the years. Despite this progress, there is still the need of earlier and more precise diagnostics and better therapeutic outcomes, since cancer remains one of the leading causes of death worldwide. In fact, over 2.6 million people will be diagnosed with cancer in Europe during 2020, with over 1.2 million expected deaths, according to the European Cancer Information System (ECIS). In Europe, the most common types of cancer for men and women are prostate and breast cancers, respectively. However, considering both sexes, lung cancer shows the highest mortality rates and accounts for the highest number of cancer deaths in Europe (>20% of the total number of cancer deaths) [1]. 

The occurrence of different types of tumours and the multifactorial etiology of cancer makes cancer an extremely complex and heterogeneous disease, where every patient develops almost a unique expression of biomarkers. For this reason, the development of the so-called precision and personalized medicine is essential to achieve better diagnostic and therapeutic outcomes. The combination of nuclear medicine modalities with nanotechnology offers unique opportunities to achieve this goal by allowing the easy and convenient merge of a variety of diagnostic and therapeutic capabilities into a single agent, within a theranostic approach of cancer. This requires the design of radiolabeled nanoconstructs that can be tailored ideally to the needs of every patient by selecting the appropriate nanoparticle, targeting biomolecule and imaging or therapeutic radionuclide [2,3,4].

Nanoparticles can be obtained with a wide variety of different materials including inorganic compounds or organic polymers, among others. The use of various materials endows the nanoparticles with a variety of morphological and physico-chemical properties, which in many cases are relevant for biomedical applications [5]. Among the different classes of nanoparticles (NPs), gold nanoparticles (AuNPs) have gained high prominence in the biomedicine field. The success of AuNPs is due to their own physico-chemical properties that are suitable for different imaging or therapeutic uses, versatile structural modification, including easy functionalization of their surface with different chemical entities (e.g., chelators, targeting biomolecules or cytotoxic drugs), favourable biological half-life, low toxicity and biocompatibility [6]. 

The favourable features of AuNPs prompted the study of their radiolabelling with a plethora of imaging and therapeutic radionuclides. A significant part of these studies intended to contribute for the design of (nano)radiopharmaceuticals for imaging and therapy of cancer. However, many of them just used the radiolabel for a more straightforward assessment of the biodistribution and pharmacokinetics of the AuNPs or for image-guided delivery of cytotoxic anticancer drugs. This comprised also image-guided biodistribution and pharmacokinetisc studies of boron cage-containing AuNPs for boron neutron capture therapy (BNCT). Having this in consideration, this manuscript provides a comprehensive review on the more recent achievements reported for radiolabelled AuNPs as nanotools for imaging and therapy of cancer. In this introductory section, the more relevant characteristics of AuNPs for their use in biomedical applications are discussed and the properties of medical radionuclides and the capabilities of the different nuclear medicine modalities are presented. 

### 1.2. Gold Nanoparticles for Biomedical Applications

Nanotechnology is a discipline of science and engineering that has led to innovative approaches in many areas of medicine based on the use of biocompatible nanoparticles. Its applications in the screening, diagnosis, and treatment of disease are collectively referred to as “nanomedicine”, an emerging field that has demonstrated great potential to revolutionize individual and population wide health in the future. It can be seen as a refinement of molecular medicine, integrating innovations in genomics and proteomics on the path to a more personalized medicine [7,8].

For biomedical applications, nanoparticles can be obtained with a wide variety of materials including inorganic compounds or organic polymers, among others. The use of different materials provides nanoparticles of different sizes and shapes with varied physico-chemical properties well-fitted for a specific use in biomedicine [9]. In this respect, it is important to have in mind the influence of surface and quantum effects that affect the chemical reactivity of nanosized materials, as well as their mechanical, optical, electric and magnetic properties [10,11]. 

**Figure 1:**
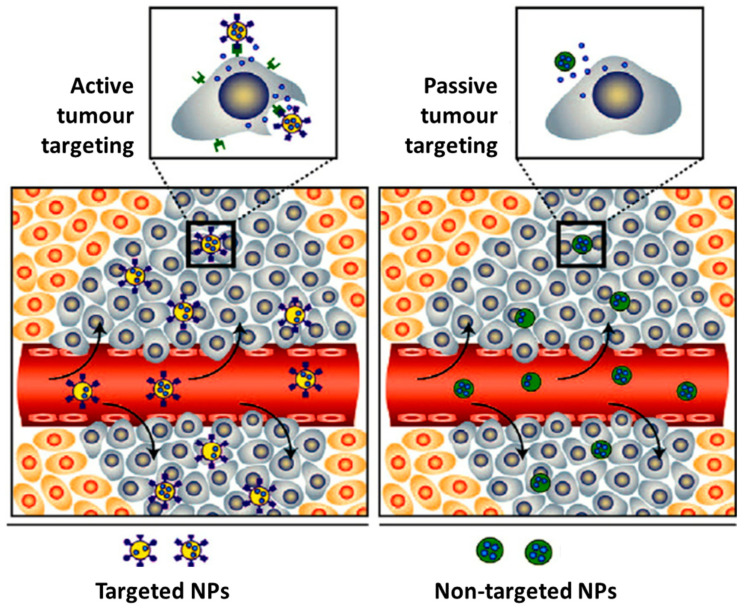
Illustration of the accumulation of nanoparticles in tumour tissues: passive vs active targeting. Adapted from Mahmoudi et al. (2011) [18].

The biological fate and potential toxicity of nanoparticles are also crucial issues, which might restrict their use for medical applications. In fact, for some of them (e.g., quantum dots), their inherent toxicity is a potential drawback but for many others (e.g., iron oxide and AuNPs) toxicity issues are less relevant. Nanoparticle biodistribution can vary greatly depending on the type and size of the particle, as well as on their surface chemistry [12,13]. For imaging and/or therapy of cancer, the selective delivery of drugs or radionuclides into the tumour tissues is of paramount importance. For this purpose, nanoparticles offer unique advantages. In fact, many NPs undergo the enhanced permeability and retention (EPR) effect that is involved in the passive targeting of leaky tumour tissues. The EPR effect is a result of the leakiness of the newly forming blood vessels and poor lymphatic drainage in growing tumours. During the angiogenesis process, the endothelial cells from the blood vessel walls do not seal tightly against each other, leaving fenestrations of approximately 200–800 nm in diameter. These processes lead to a passive accumulation of nanoparticles in tumour tissues, as shown in Figure 1 [14]. On the other side, the versatile functionalization of the NPs surface with targeting biomolecules (e.g., a peptide or an antibody) allows the specific targeting of tumours through interaction with receptors overexpressed in the tumour cells or in the tumour microenvironment (Figure 1) [15,16,17].

**Figure 2:**
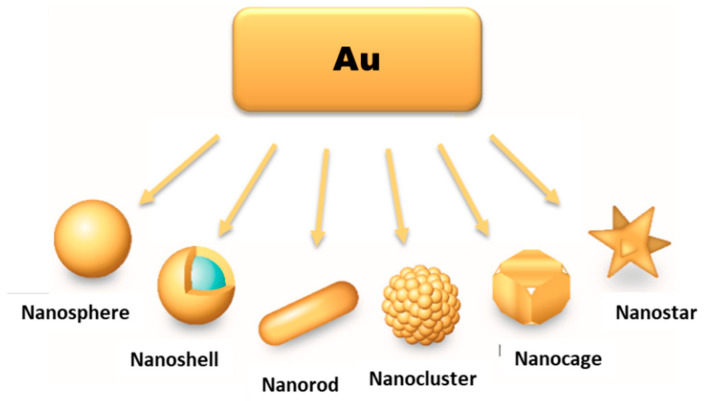
Different types of AuNPs, according to their shape and morphology. Adapted from L. F. de Freitas et al. (2018) [21].

For biomedical applications, namely for cancer imaging and therapy, AuNPs offer the possibility of a versatile functionalization with targeting biomolecules for specific accumulation in tumour tissues, allowing more precise diagnostics and/or localized therapeutic effects. Moreover, there are currently available different methods to manipulate the size and shape of gold nanoparticles, spanning from shapes like nanospheres (or nanoshells), nanorods, nanocages to nanostars (Figure 2), to obtain AuNPs tailored to the different biomedical uses [19,20]. 

### 1.3. Nuclear Medicine Modalities and Medical Radionuclides

Nuclear medicine procedures involves the administration of radiolabeled drugs that are called radiopharmaceuticals, which are used for diagnostic or therapeutic applications depending on the physical properties of the labeling radionuclide.

**Figure 3:**
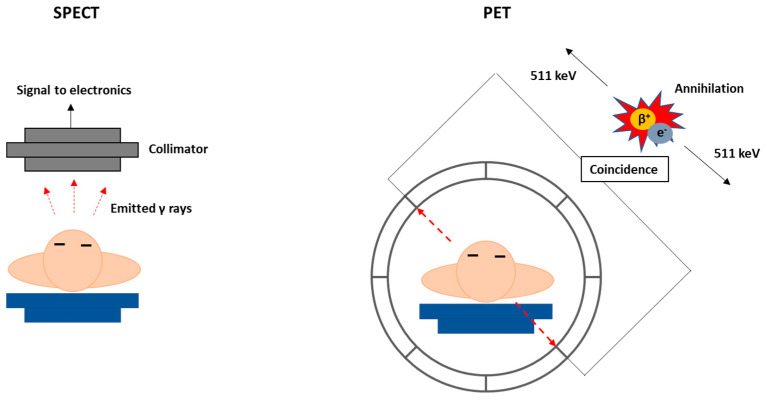
Schematic diagrams of SPECT (**left**) and PET (**right**) imaging.

The two fundamental nuclear medicine imaging techniques are single-photon emission computed tomography (SPECT) and the positron emission tomography (PET) (Figure 3). Currently, SPECT and PET scans are essential for the diagnosis and follow-up of patients and can provide unique biological information, at molecular level, on healthy and pathological processes. By contrast, other imaging modalities, such as magnetic resonance imaging (MRI) or computed tomography (CT), only provide anatomical images or functional data. Nowadays, multimodal devices such as PET-CT, SPECT-CT or PET-MRI can combine in a synergic manner these techniques providing images with both quantitative functional information and high-resolution anatomic reference [22]. The high sensitivity of nuclear imaging techniques allows the detection of the photons emitted by the radiopharmaceuticals administered systemically, usually in an intravenous manner, to evaluate organ functionality and disease progression. Contrarily to the contrast agents used in other imaging techniques such as MRI or CT, the sub-nanomolar range dosage of radiopharmaceuticals does not induce any biochemical alteration in the system that is being imaged. Biochemical alterations always occur before anatomical changes. Therefore, PET and SPECT are more adequate for molecular imaging applications and earlier diagnostic of disease, when compared with classical CT, MRI or ultrasound (US) imaging. Nonetheless, it is important to notice that recent progresses in the development of more sensitive target-specific contrast agents for MRI or US imaging might render these techniques with higher translational potential for diagnostic molecular imaging [23]. However, nuclear imaging techniques offer the unique advantage to easily switching from a diagnostic radionuclide to a therapeutic one, using the same chemical entity, giving rise to an increasing number of clinical applications with theranostic radiopharmaceuticals, as detailed below.

Radionuclides useful for imaging emit either γ-photons or positrons and their optimal half-life is generally going from some minutes to few hours, as can be seen in Table 1. SPECT imaging involves the detection of γ-photons in a gamma camera placed outside the patient, which are emitted directly by the radionuclide with an energy typically in the range 100–250 keV. PET imaging is based on the detection of back-to-back 511 keV annihilation photons that result from the interaction of the positrons emitted by the radionuclide with electrons from the surrounding medium (Figure 3) [24,25]. 

The radionuclides used in therapy are generally α or β^−^ emitters. However, the cytotoxicity mediated by low-range Auger electrons, emitted by radionuclides undergoing electron capture (EC) and internal conversion (IC) decay processes, have also gained considerable attention when properly delivered to tumour cells [26]. All these radionuclides emit particulate radiation with different path-lengths and linear energy transfer (LET) values in soft tissues, allowing to choose the best suited for the specificity of the disease to target. Some of the most relevant radionuclides useful for therapy are presented in Table 2.

In current nuclear medicine practice, therapeutic approaches using radionuclides are still limited to the treatment of radiosensitive tumours, being generally preferred other strategies such as surgery, external radiotherapy or conventional chemotherapy for the treatment of solid malignancies. However, the possibility of integrating imaging and therapy make radiopharmaceuticals powerful tools for the development of more personalized approaches, especially in cancer theranostics. The term theranostics accounts for the almost unique opportunity that radiopharmaceuticals offer to develop more specific, individualized therapies and to combine diagnostic and therapeutic capabilities into a single agent. The same targeting biomolecule recognizing a particular molecular target, can be labelled either with a diagnostic or with a therapeutic radionuclide allowing the development of a patient-specific treatment [27]. For example, significant progresses have been reported recently for somatostatin analogs labelled with ^68^Ga for PET imaging or with ^177^Lu for peptide receptor radionuclide therapy (PRRT). These progresses led to the approval of the radiopharmaceuticals ^68^Ga-DOTATATE (NETSPOT^®^) and ^177^Lu-DOTATATE (LUTATHERA^®^) for clinical use in the diagnosis and treatment of neuroendocrine tumours (NETs) mediated by somatostatin receptor, both in Europe and in the USA [28]. 

As reviewed herein, AuNPs were evaluated in several instances as delivery systems for some of the medical radionuclides that are presented in Table 1 and Table 2. Part of the reported research work aimed at the design of innovative (nano)radiopharmaceuticals for imaging and therapy of cancer [29]. However, many of these studies dealt with AuNPs labeled with imaging radionuclides just to achieve a more straightforward evaluation of their in vivo biological fate and pharmacokinetics, profiting from the non-invasiveness and high sensitivity inherent to nuclear imaging modalities. For this purpose, it is of great importance that the incorporation of the radioisotope remains stable under in vivo conditions in order to exert properly its function. Otherwise, radioisotope biodistribution will no longer reflect that of the nanoparticles, meaning that the imaging data will not be useful to assess the fate of the nanoparticles. 

## 2. Synthesis of Gold Nanoparticles

**Figure 4:**
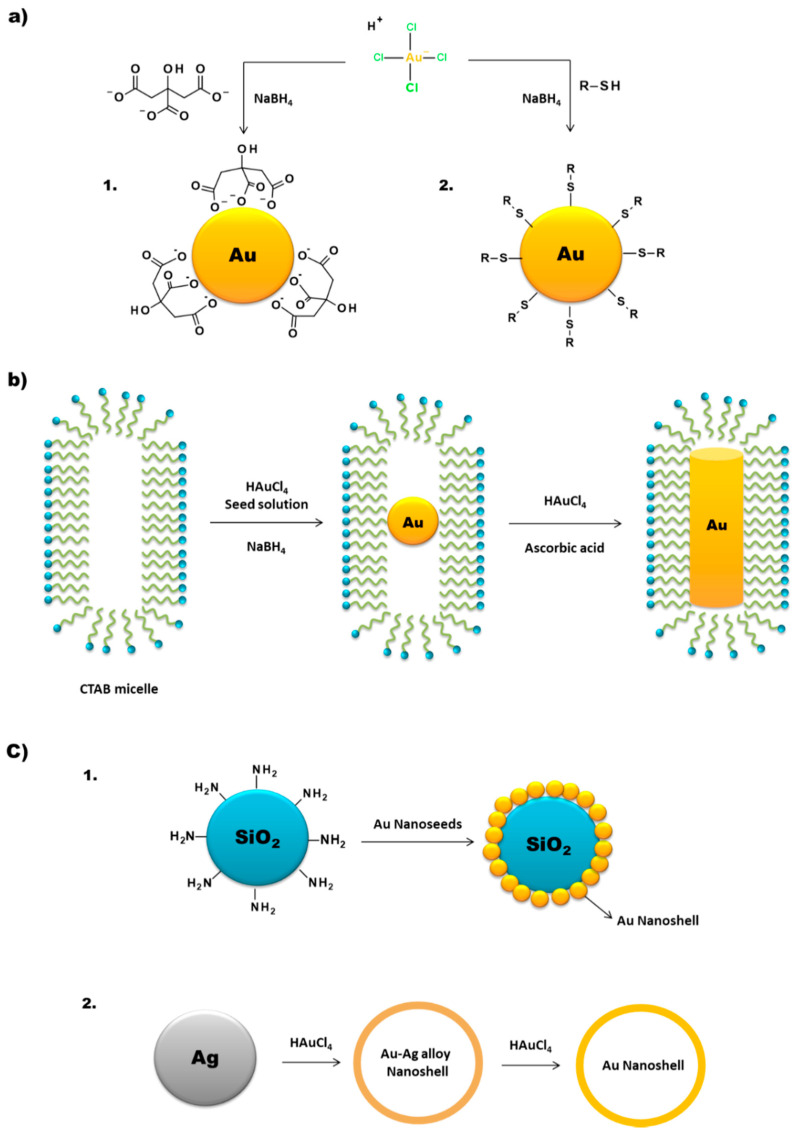
Schematic synthesis of (**a**) AuNPs by the (1) Turkevitch and (2) Brust methodologies, (**b**) AuNRs by the seed-mediated method, and (**c**) (1) core and (2) hollow AuNSs.

One of the most common methods of AuNP synthesis is by reduction of a gold precursor, generally the tetrachloroauric acid (HAuCl_4_), in the presence of a stabilizing agent (Figure 4a). In order to guarantee the reduction of the gold, strong to mild reducing agents are used, like NaBH_4_, hydrazine or citrate. In 1951, Turkevitch et al. developed one of the most conventional synthetic routes, still in use to this day, which consists on the reduction of Au(III) in HAuCl_4_ by citrate in water. It is known as the citrate reduction method, which allows the formation of citrate stabilized AuNPs and a controlled size of the particles by varying the citrate/gold ratio [30]. A few years later, in 1994, Brust et al. introduced a new procedure for the efficient synthesis of stable AuNPs with reduced dispersity and controlled size, which represented at the time an important breakthrough. This procedure is based on the use of thiolated ligands that strongly bind to gold due to the soft character of both Au and S. After addition of a reducing agent (NaBH_4_), the Au(III) is reduced to Au(I) and the AuNPs are formed [31]. This opened the opportunity to develop AuNPs using a great variety of thiolated ligands. This method allows the control of core nanoparticle size by shifting the ratio of thiol/Au in the reaction mixture; for instance, the use of larger thiol/Au ratios affords smaller core sizes with less polydispersity [32,33].

In recent years there has been an increased interest on green methodologies for the synthesis of AuNPs, using alternative reducing agents to NaBH_4_ or hydrazine that are environmentally toxic. In this regard, Katti et al. have developed extensive work with phytochemical agents extracted from various biological media (e.g soybeans, tea leaves) [34,35,36]. It was demonstrated that these phytochemical agents performed the dual function of reducing the gold salt to form the AuNPs and at the same time provide a protein coating that can stabilize the nanoparticle structure [35,36].

As mentioned above, AuNPs can be obtained in various forms, including nanospheres, nanorods, nanoshells or nanocages. The synthetic methods described above are commonly used to obtain AuNPs in spherical amorphous form. The synthesis of AuNPs with a more complex shape requires alternative methodologies [6,21,37]. Gold nanorods (AuNRs) are commonly synthesized through the seed-mediated approach, which involves a two-step process where initially a seed solution is prepared with tetrachloroauric acid in the presence of a strong reducing agent (e.g., NaBH_4_) (Figure 4b). The seed solution is then added to a mixture of cetyltrimethylammonium bromide (CTAB), a mild reducing agent (e.g., ascorbic acid) and tetrachloroauric acid. The elongated ellipsoidal shape of the CTAB micelles permits the growth of the AuNPs of the seed solution in an elongated manner, in order to obtain a rod shape [38,39,40]. Some variations on this procedure include the addition of AgNO_3_ prior to the growth phase, which allows a better control of the shape and increase the yield of AuNRs [38]. 

Besides the seed-mediated method, other methodologies have also been reported in literature for the synthesis of AuNRs. The template method is based on the electrochemical deposition of Au within nanoporous template membranes, which can be of different materials (e.g., polycarbonate or alumina). Ag or Cu is added to the template membrane to form a conductive film that allows for the electrodeposition of Au and growth of the nanoparticles within the membrane nanopores. The nanorods are then recovered by selective dissolution of the template membrane and Ag or Cu film. The diameter of the AuNRs is dependent of the nanopore diameter of the membrane, while the length can be controlled by the amount of Au deposited [41,42].

Electrochemical methods for AuNR synthesis are usually based on the use of a dual electrode electrochemical cell. A gold layer is used as the anode and a platinum layer as cathode. Both electrodes are immersed in a surfactant solution composed of the cationic surfactant CTAB and a more hydrophobic cationic surfactant like tetradodecylammonium bromide (TCAB), which are responsible for the formation of the rod-shaped nanoparticles. During the process of controlled current electrolysis, the gold layer releases Au ions that migrate to the cathode where reduction occurs and the AuNRs are formed [43].

Gold nanoshells (AuNSs) can be of two types, namely solid or with a hollow core (Figure 4c). The synthesis of core-containing AuNSs is based on the use of a seed nanoparticle, which will form the core of the nanoshell. Then, the addition of tetrachloroauric acid in the presence of a reducing agent leads to the deposition of gold seeds on the surface of the core. SiO_2_ is one of the most commonly used cores. These silica nanoparticles have a capping agent on their surface, like 3-aminopropyltriethoxysilane (APTES), which provides NH_2_ groups that can link to the gold [44].

For the synthesis of hollow AuNSs, one approach is to use the silica core to synthesize the gold nanoshells as described above and then use HF to remove the SiO_2_ core. Another method is the template galvanic replacement of silver. This methodology is based on the higher standard reduction potential of the AuCl_4_^−^/Au pair when compared with that of the Ag^+^/Ag pair. Silver is oxidized into Ag^+^ when silver nanostructures and HAuCl_4_ are mixed in an aqueous medium. By optimizing the ratio between the silver nanoparticles and HAuCl_4_, silver atoms can diffuse into the gold shell (or sheath) to form a seamless, hollow nanostructure with its wall made of Au-Ag alloys [44,45]. The further increasing of the HAuCl_4_ present in the medium triggers a dealloying process that selectively removes silver atoms from the alloyed wall. This induces morphological reconstruction that leads to the formation of pinholes in the walls, and the nanoparticles acquire a cage like structure. This is one of the common methodologies for the synthesis of gold nanocages (AuNCs). Temperature also plays an important role in the replacement reaction because the solubility constant of AgCl and the diffusion coefficients of Ag and Au atoms are both strongly dependent on this parameter [45].

Due to the inherent difficulties in analyzing nanoscale materials, in comparison with molecular or bulk materials, the characterization of NPs requires particular analytical techniques and methodologies. It is common to recur to various characterization techniques, in a complementary manner, to obtain reliable information on the NPs structure and their physico-chemical properties. Besides the techniques summarized below, there are various other methodologies available nowadays for NP characterization. The use of a single one of these characterization techniques cannot provide all the required data for a proper assessment of the NP structure, hence it is necessary to take into consideration the technique’s strengths and weaknesses, depending on the nature of the NP [46,47].

Microscopy techniques, like transmission electron microscopy (TEM) or scanning electron microscopy (SEM), can provide information regarding the size and shape of the nanoparticles. On the other hand, the study of the hydrodynamic size distribution relies on techniques like dynamic light scattering (DLS) or nanoparticle tracking analyses (NTA), which can also provide information on the agglomeration state of the NPs in solution. Other commonly used techniques are zeta-potential measurements for surface charge determination and UV-Vis spectroscopy for characterization of optical properties, namely to determine the surface plasmon resonance wavelength that can be correlated with the size and shape of the nanoparticles. In the particular case of metallic NPs, X-ray-based techniques, like X-ray diffraction (XRD), are used to assess the crystalline structure and elemental composition [46,47].

## 3. Radiolabelling of Gold Nanoparticles

To pursue with a stable radiolabeling of AuNPs it is commonly required to perform their functionalization with suitable molecular entities, which will allow for the coordination/conjugation of the radioisotopes [48]. In this regard, there are different synthetic pathways available to functionalize AuNPs: (i) using bifunctional molecules that can act as a capping/stabilizing agent during the synthesis of the AuNPs and that can bind to the radioisotopes [49,50]; (ii) direct conjugation of amino/thiolated molecules to the surface of preformed AuNPs [51,52]; (iii) ligand exchange, in which some/all of the capping/stabilizing molecules on the AuNPs are exchanged with a different molecule with gold bonding capabilities [53]; and (iv) chemical modification of molecules already present in the AuNP structure [54,55].

**Figure 5:**
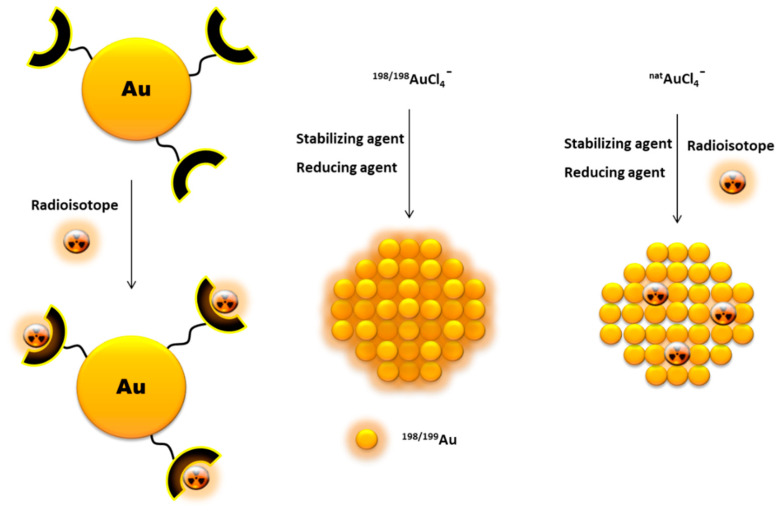
Schematic drawing of different pathways to incorporate radionuclides into AuNPs.

Another way to incorporate radionuclides into the AuNP structure, without their further chemical functionalization, is by directly introducing the radioisotopes in the nanoparticle core (Figure 5). This is commonly achieved by using a ^198/199^Au precursor in the synthesis of the nanoparticles [56,57]. Alternatively, it has also been reported the neutron irradiation of non-radioactive AuNPs to originate ^198/199^Au-containing nanoparticles through neutron capture reactions (^197^Au(n,γ)^198^Au and ^198^Au(n,γ)^199^Au) [58].

In some cases, it is possible to attach other radionuclides to the AuNPs without the need of extra chemical derivatization. This can be achieved by adsorption of the radionuclide to the AuNP surface, namely for ^131^I or ^64^Cu [59,60]. The incorporation of the radionuclide in the NPs core is another possibility, as reported by Liu et al. for ^64^Cu alloyed AuNPs modified with PEG. These ^64^Cu-labeled AuNPs were obtained starting from HAuCl_4_ and ^64^Cu(acac)_2_ and using oleylamine as reducing agent [61]. In the same way, Chen et al. have studied the integration of a ^64^Cu shell into PEG-stabilized AuNPs by reducing ^64^Cu(II) in the presence of hydrazine and polyacrylic acid [62].

## 4. Examples of Radiolabeled AuNPs for Nuclear Imaging Applications

As summarized in Table 3, many imaging radionuclides were used in the labeling of a variety of AuNPs to evaluate their biological fate in selected cellular and animal models, which included ex-vivo biodistribution studies or nuclear imaging scans (PET or SPECT). Part of this work also involved studies of image-guided drug delivery by AuNPs. In the next sub-sections, the most recent and relevant results are reviewed by the types of radionuclides used, i.e., radiohalogens vs. radiometals.

### 4.1. Radiohalogens

#### 4.1.1. Fluorine-18 (^18^F)

Fluorine-18 (^18^F) is the most widely used positron emitter in clinical PET imaging [103,104]. Conversely, there are only few examples of gold nanoparticles radiolabeled with ^18^F aiming at their assessment as PET probes. Kogan and co-authors were the pioneers in the biological evaluation of gold nanoparticles radiolabeled with ^18^F. In 2012, they attached covalently [^18^F]-fluorobenzoate to gold nanoparticles. The nanoparticles were functionalized with the Cys-Leu-Pro-Phe-Phe-Asp (CLPFFD) peptide, which has potential use in the treatment of Alzheimer disease by removing the toxic β-amyloid aggregates formed, and with the Cys-Lys (CK) peptide, which allows conjugation of the N-succinimidyl-4-[^18^F]-fluorobenzoate ([^18^F]-SFB) through the reaction of the amine of the side chain of the amino acid K with the carbonyl function present in the [^18^F]-SFB [104]. Biodistribution studies, performed two hours after intravenous administration of the resulting ^18^F-labeled nanoconjugate in rats, have shown high accumulation of radioactivity in the bladder and urine due to the peptide-associated pharmacokinetics. Lungs, liver, intestine, kidneys, blood are also target organs, being observed the lowest uptake of radioconjugate in the pancreas and brain [63]. Aiming to overcome the small uptake of radiolabeled AuNPs in the brain, Schirrmacher et al. assessed the properties of new gold nanoparticles bearing a maleimide group, partially hydrolyzed and non-hydrolyzed, and the prosthetic silicon-fluorine group ^18^F-SiFA-SH [64]. Brain images obtained by in vivo micro PET scans of normal rats, at 2 h after intravenous injection of the ^18^F-labeled AuNPs, revealed a higher brain uptake of the partially hydrolyzed form (0.13% ID/g) relatively to the non-hydrolyzed congeners (0.07% ID/g). As proof-of-concept, the authors functionalized these partially hydrolyzed radio-gold nanoparticles with a cysteine derivative of the octreotate peptide TATE, which has a high appetency for the somatostatin receptors present in several endocrine tumours. MicroPET biodistribution studies showed that the target-specific AuNPs have a similar brain uptake as the starting radio-nanogold platform partially hydrolyzed [64,65].

#### 4.1.2. Iodine-124 (^124^I)

Iodine-124 is a rather-long lived positron emitter (T_1/2_ = 4.18 d) that is very suitable for the radiolabeling of compounds with long circulation times and/or slow excretion rates, as is often the case of AuNPs [105]. Lee and co-authors developed AuNPs functionalized with tannic acid (TA-AuNPs), which were radiolabeled with ^124^I and subsequently surrounded by a protective shell of Au to obtain the final NPs (^124^I-TA-Au@AuNP). ^124^I-TA-Au@AuNP was evaluated as a PET probe to label dendritic cells (DCs) and visualize their migration to lymphoid organs [105]. DCs can recognize several types of tumour-specific or associated antigens and induce anti-tumour immune reactions [106]. In vivo PET/CT images of mice, subcutaneously injected into the footpad with bone marrow-derived DCs (BMDCs) labeled with ^124^I-TA-Au@AuNPs, showed that the cells predominantly migrate to the draining lymph nodes. When the mice were pre-conditioned with tumour necrosis factor alpha (TNF-α), the ^124^I-TA-Au@AuNPs-BMDCs could be detected in the popliteal lymph nodes, after 15 h injection and until 96 h post injection [106]. The same authors have also performed studies with radionuclide-embedded AuNPs, carrying DNA (^124^I-RIe-AuNPs), PEG (^124^I-PEG-RIe-AuNPs) and polypeptides (^124^I-Poly-Y-RIe-AuNPs) [106]. The in vivo PET/CT and Cerenkov luminescence imaging (CLI) images obtained after injection of RIe-AuNPs into the foot pad of mice showed a highly selective migration of the labeled DCs to draining popliteal lymph nodes (DPLNs). Additionally, the combined in vivo PET/CLI images obtained for rats administered with ^124^I-PEG-RIe-AuNPs demonstrated that the AuNPs are effectively captured by the sentinel lymph nodes [107]. The CLI in vivo images also showed strong optical signals in lung, liver and spleen, with image quality equivalent to that of PET/CT images. Ex-vivo biodistribution studies have confirmed the migration of DCs to DPLNs, when in general optical imaging cannot detect the migration of DCs to deep tissues [106]. RIe-AuNPs have also demonstrated capabilities to monitor macrophage migration and, therefore, to follow-up the therapeutic effects of anti-inflammatory agents in vivo by PET imaging [95]. Studies with Poly-Y-RIe-AuNPs have shown that this platform not only allows selective screening of migration from DCs to lymphoid organs, but also promotes maturation of DCs with production of significant amounts of cytokines, such as TNFα and IL-6, in the spleen and lymphatic drainage nodes. PEG-RIe-AuNPs were evaluated as imaging probes for the detection of sentinel lymph nodes. The combined PET/CLI in vivo images performed on rats clearly demonstrated that PEG-RIe-AuNPs are effectively captured by the sentinel lymph nodes [96].

The same authors have also developed crushed gold shell radioactive nanoballs (^124^I-Au@AuCBs) and assessed their theranostic potential in photothermal therapy, based on a macrophage-mediated delivery of the NPs to the tumour tissues. The authors demonstrated the capability of ^124^I-Au@AuCBs to enhance photodynamic therapy in colon cancer bearing mice, when administered intratumourally [97,99]. The authors have also designed pegylated ^124^I-Au@AuCBs, which have been evaluated for multimodal (PET/CLI) in vivo detection of sentinel lymph nodes. The lymph nodes could be detected in mice following subcutaneous injection into the footpad, and its accumulation persisted until 24 h post injection. However, the utility of the platform as a lymphatic tracer is hampered by its unexpected in vivo toxicity [96,98].

#### 4.1.3. Iodine-125 (^125^I)

Iodine-125 emits gamma rays followed by an average of 21 Auger electrons per decay with very low energies (0.050–0.500 keV). ^125^I is used clinically for brachytherapy, namely as ^125^I-seeds to treat prostate cancer, and has been thoroughly investigated at preclinical level for Auger therapy of cancer [26]. ^125^I is not the best suited radionuclide for imaging applications but it can be used for biodistribution studies and even for SPECT imaging scans. For this reason, ^125^I has been used in several instances to radiolabel AuNPs and to image their biodistribution in animal models [108]. For instance, Zhang and co-workers have recently reported on cisplatin-loaded and ^125^I-labeled gold nanoparticles (RGD-^125^IPt-AuNPs and RGD-^125^IPt-AuNRs) carrying an arginine-glycine-aspartic acid (RGD) peptide analog. These RGD-containing gold NPs were evaluated for their tumour accumulation and chemo-radiotherapy efficacy in mice xenografts. In vitro studies, performed on the human derived α_v_β_3_ positive H1299 cells, have shown that both types of nanoparticles exhibit high affinity and specificity for α_v_β_3_. However, SPECT/CT imaging of H1299 tumour xenograft nude mice, intravenously injected with the ^125^I-labeled AuNPs, demonstrated that tumour accumulation of the rod-shaped RGD-^125^IPt-AuNRs was significantly higher than that of the spherical RGD-^125^IPt-AuNPs, at each time point. However, no significant difference was observed for the distribution patterns of these two types of probes in the other major organs, such as the liver and spleen [99]. 

### 4.2. Radiometals

#### 4.2.1. Copper-64 

Copper-64 (^64^Cu) is one of the most widely used PET radioisotopes for nanoparticle labeling. Accordingly, several types of ^64^Cu-gold nanoparticles have been reported for the development of cancer theranostic tools based on PET imaging. The ^64^Cu radiolabeling was achieved either through chelator-free or through chelator-based strategies [109].

Using the chelator free approach, Xie et al. have studied the ^64^Cu-radiolabeling of gold nanoshells functionalized with a RGD peptide derivative (^64^Cu-NS-RGDfKs). The biodistribution and tumour specificity of the ^64^Cu-NSs were assessed by PET-CT imaging of live nude rats xenografted with head and neck squamous cell carcinoma (HNSCC). The images showed that the integrin-targeted ^64^Cu-NS-RGDfKs have a higher concentration in the tumour than the non-targeted ^64^Cu-NS-PEG, although they have similar biodistribution trends. Post-mortem biodistribution analyses by measurement of radioactivity (^64^Cu) and NAA (gold content), 46 h after intravenous injection, confirmed the improved tumour accumulation of the targeted NSs. In addition, the usefulness of the NS-RGDfKs as a photothermal therapeutic enhancer agent was confirmed in treatments conducted in nude mice xenografts with subcutaneous HCT116 human colorectal cancer [66].

Liu and co-authors developed spheric AuNPs radiolabelled with ^64^Cu, in which the radioisotope was incorporated directly into the structure of the AuNP core. These nanoparticles are very stable and constitute a good platform for oncology PET imaging, as shown by in vivo studies in rats bearing EMT-6 breast carcinoma. The studies showed that the tumour can be clearly visualized by these ^64^Cu-containing AuNPs with a definition similar to that obtained with ^18^F-2-deoxyglucose (^18^F-FDG), at 1 h p.i. [61]. Subsequently, other ^64^Cu-containing AuNPs with different shapes and sizes have been synthesized by a similar methodology. In particular, PEG modified gold nanorods (Au NR) and decorated with a RGD peptide analog, with UV absorption around 808, were radiolabeled with ^64^Cu. The resulting radioactive AuNRs (RGD-[^64^Cu]Au NR808) showed good potential for cancer theranostics, namely for PET image-guided photothermal therapy [62]. In fact, in vivo PET imaging studies performed in U87MG tumour xenograft rats, injected intravenously with RGD-[^64^Cu]Au NR808, clearly showed the accumulation of the NPs in the liver (21.7% ID/g, 45 h p.i.), spleen and tumour (7.6% ID/g, 45 h p.i.), at early and late post-injection times. Quantitative ROI analysis showed that the maximum tumour uptake of these radiolabeled AuNR was reached at 24 h post injection (8.37% ID/g), which stayed above 7% ID/g even after 45 h of administration. Xenograft rats irradiated with laser after injection with RGD-[^64^Cu]Au NR808 showed a remarkable decrease in tumour growth after two days of treatment. In addition, it was observed an insignificant tumour recurrence after 8 days of combined treatment. On the opposite, a clear growth of the tumour was observed in rats submitted only to laser treatment [62].

Within the chelator-based strategy, 1,4,7,10-tetraazacyclododecane-1,4,7,10-tetra-acetic acid (DOTA) derivatives are the most common choice for the ^64^Cu-labeling of AuNPs. Hollow gold nanospheres containing, or not, a RDG peptide derivative were radiolabeled with ^64^Cu via a thioctic acid-PEG-DOTA derivative. In order to enhance the uptake in liver tumours, the HAuNSs without the RGD peptide were coated with iodized oil (lipiodol). The tumoural uptake of the resulting NPs (^64^Cu-PEG-HAuNS-lipiodol) was evaluated in rabbits bearing hepatic VX2 tumours, after intravenous (i.v.) and hepatic intra-arterial (i.a.) injections and using PET/CT imaging. These studies showed that the retention of the HAuNSs is highly dependent on the route of administration, being the highest tumoural uptake achieved with i.a. Moreover, ^64^Cu-PEG-HAuNS-lipiodol presented a tumour uptake almost 4 times superior than the observed for the congeners without lipiodol and 2.5 times superior than the HAuNSs decorated with a RGD peptide. No significant difference was observed for the tumoural uptake of ^64^Cu-PEG-HAuNS and ^64^Cu-RGD-PEG-HAuNS administered intravenously [68].

The group of Tam et al. also reported on DOTA-containing AuNSs radiolabeled with ^64^Cu and on their evaluation in ablative treatments of rabbits with hepatic VX2 tumours [68]. The rabbits were subjected to different ablative treatments: nanoembolization (NE) alone and in combination with radiofrequency ablation (RFA+NE), irreversible electroporation (IRE+ NE) and laser induced thermal therapy (LITT+NE). NE was performed with ^64^Cu-DOTA-hollow-gold nanoparticles loaded with doxorubicin, which is the chemotherapeutic agent most frequently used in hepatic cancer therapy. PET images, obtained 1 and 18 h after each treatment, showed a great dependence on the location and accumulation of radionanoparticles with time and with the ablative energy applied in the treatment. The IRE + NE treatment resulted in the deposition of nanoparticles in and around the tumoural liver cells, enhancing the possibility to determine a more precise ablation zone by PET imaging [67].

Liu and co-authors evaluated the pharmacokinetics and tumour uptake of gold nanocages ^64^Cu-DOTA-PEG-AuNCs (30 and 55 nm) using in vivo PET/CT imaging. In normal male C57BL/6 mice, the 30 nm-^64^Cu-DOTA-PEG-AuNCs showed the best in vivo profile, with high blood, lung and heart retention and reduced reticuloendothelial system (RES) uptake. The biodistribution profile obtained for these AuNCs in nude mice bearing EMT-6 breast cancer is analogous. The tumour uptake quickly increases overtime (2.68 ± 0.12% ID/g; 7.2 ± 0.9% ID/g; 7.9 ± 1.1% ID/g, at 1 h; 4 h and 24 h, respectively) and it is almost four times superior to that observed for the 55 nm-^64^Cu-DOTA-PEG-AuNCs. The authors claimed that this tumour retention over time is particularly important for longitudinal and repeated photothermal cancer treatments. In addition, due to the relatively fast blood clearance (blood uptake higher than 20% ID/g at 1h and less than 3% ID/g at 24 h), the ratios tumour/muscle and tumour/blood also increase considerably overtime. All these findings prompted the authors to consider these nanoplatforms as a robust tool for further research studies in cancer theranostics [73].

In vivo PET studies with ^64^Cu(-DOTA)-gold nanocages incorporating α-melanocyte-stimulating hormone (α-MSH) peptide (^64^Cu-AuNCS-PEG-MSH) enabled the very adequate imaging of tumours in mice bearing B16/F10 melanoma, at 24 h post injection. The tumoural uptake of these nanoparticles is related to the concentration of the α-MSH peptide present in their surface to target the melanocortin 1 receptor (MC1R). Maximum tumour uptake of these MC1R-targeting gold nanocages varied from 7.43 + 0.55% ID/g to 7.52 + 0.40% ID/g, at 24 to 48 h post injection. Nevertheless, studies to improve the biodistribution profile and reduce the inherent toxicity of ^64^Cu-AuNCS-PEG-MSH are desirable in order to drastically reduce the liver and the spleen uptake (approximately 2.5 and 12 times superior to the tumour uptake, respectively) [69]. 

DOTA-based complexes with several metals, namely Cu, have a high thermodynamic stability [110]. However, there are some evidences that DOTA is not the ideal chelator for ^64^Cu with possible in vivo release of the radiometal and concomitant accumulation in liver [70,111,112,113]. On the other hand, Cu-NOTA complexes (NOTA = 1,4,7-triazacyclononane-1,4,7-triacetic acid) also have similarly high thermodynamic stability in solution, but in general show superior kinetic stability in vivo compared to their Cu-DOTA counterparts. The same is verified for some other metals, like in the case of Ga [70,110,114,115].

Taking this into consideration, Pretze and co-authors focused on AuNPs functionalized with the NOTA derivative NODAGA, aiming to obtain AuNPs more stable in vivo and suitable for dual imaging of prostate cancer using near-infrared (NIR) fluorescence and PET. Towards this goal, these authors assessed the pharmacokinetics of PEGylated AuNPs carrying NODAGA as the chelating agent for complexation of ^64^Cu and decorated with a NIR dye (SIDAG). To recognize prostate cancer cells, the AuNPs were further functionalized with a bombesin (BBN) peptide analog ([7,8,9,10,11,12,13,14] BBN) or with a Lys-Urea-Glu (LUG) motif for the targeting of the gastrin releasing peptide receptor (GRPr) or the prostate-specific membrane antigen (PSMA), respectively. In vitro assays were performed for ^64^Cu-AuNP-BBN and ^64^Cu-AuNP-LUG to assess their acute and long-term toxicity in PC3 and LNCaP cancer cell lines, due to the action of the β^−^ radiation. After 24 h of incubation, the toxicity induced by the nanoparticles was higher in the LNCaP than in the PC3 cell line. This result was somewhat unexpected for ^64^Cu-AuNP-BBN, since the LNCaP cell line does not express the gastrin releasing peptide (GRP) receptor that is recognized by BBN derivatives. However, after four days of incubation, ^64^Cu-AuNP-BNN displayed higher toxicity in PC3 cells while ^64^Cu-AuNP-LUG had higher toxicity in the LNCaP cell line, as expected. Ex vivo biodistribution studies performed in healthy male SHO mouse with co-injection of ^64^Cu-AuNP-LUG and AuNP-NIR-LUG showed similar uptake of the fluorescent and radioactive AuNPs in the different organs, 25 h after injection. However, the biodistribution profile of ^64^Cu-AuNP-NIR-LUG in male athymic nude mice showed radioactivity uptake in brain, spleen, and pancreas lower than the uptake in the same organs measured based on the respective fluorescence intensities. The authors attributed this discrepancy to the possible release of the NIR dye in vivo [70].

Other less common types of chelators have also been explored for the ^64^Cu-labeling of AuNPs. The bicyclam plerixafor (AMD3100) chelator was used to stabilize a gold nanocluster (^64^CuAuNCs−AMD3100) that showed high and improved stability. AMD3100 is a CXCR4 antagonist approved for the mobilization of hematopoietic stem cells in lymphoma and multiple myeloma patients, under the trademark Plerixafor. ^64^CuAuNCs−AMD3100 was evaluated as a PET radioprobe to detect in vivo the expression of the chemokine receptor CXCR4 in a 4T1 mouse orthotopic breast cancer model with lung metastases, through PET imaging. It was found a strong correlation between the CXCR4 receptor levels in the tumour and the quantitative tumoural uptake of ^64^CuAuNCs−AMD3100. Moreover, competitive receptor blocking studies confirmed a tumour accumulation mediated by the CXCR4 receptors. Taking together all these findings, the authors claimed that these radio nanoclusters showed a good potential in translational research for the first early cancer and metastasis diagnosis. However, these excellent results were not translated into later phases of primary and metastatic breast cancer. Some improvements still need to be made so that ^64^CuAuNCs—AMD3100 extend its potential usefulness for the diagnosis of breast cancer and its metastasis, in all stages of the disease [71].

#### 4.2.2. Gallium-67/Gallium-68 

^67^Ga is a gamma emitter suitable for SPECT imaging while ^68^Ga is an emerging PET radionuclide (Table 1). For this reason, the evaluation of ^67/68^Ga compounds as medical diagnostic probes, carried out over the past few decades, has been a very active field of research. Contrastingly, the evaluation of nanoparticles labeled with these radioisotopes has been much less intense, namely when compared with ^64^Cu [49,116,117]. 

As mentioned above, Pretze et al. have evaluated ^64^Cu-AuNP-BBN and ^64^Cu-AuNP-LUG as new nanotools for the theranostic of prostate cancer. In the same work, these authors have also extended their studies to the ^68^Ga-labelled congeners. It was observed that ^68^GaAuNPBBN has a strong internalization in prostate cancer PC3 cells, within 3–5 h of incubation, being mainly concentrated in the cytoplasmic fraction. Blockade experiments performed in PC3 and in LNCaP cell lines with monomeric BBN (7–14) showed a significant reduction in the cellular internalization of ^68^GaAuNPBBN. Analogous results were observed in blockade experiments with LUG for ^68^GaAuNPLUG in LNCaP cells. These findings led the authors to conclude that the cellular uptake of these nanoparticles involves, at least in part, a receptor-specific mechanism [77].

Silva and co-workers have studied spherical AuNPs stabilized with thiolated derivatives of DOTA or DTPA (diethylenetriaminepentaacetic acid), proceeding with their ^67^Ga labeling and their preclinical evaluation in cellular and animal models of prostate cancer. Initial in vitro studies indicated that the DOTA-containing AuNPs display a higher capability to maintain the radiometal coordination than the DTPA congeners, in the presence of various media or biological substrates [49]. The AuNP-DOTA nanoparticles were decorated with BBN analogs, covalently appended by a unidentate cysteine or a bidentate thioctic group to form the nanoconstructs CBBN-AuNP-TDOTA and BBN-AuNP-TDOTA, respectively. Competitive binding assays in prostate cancer PC3 cells showed that both nanoconstructs have a high affinity towards the GRPr; however, there was a significant contrast in the cell internalization behavior of the two radiolabeled nanoconstructs in the same cell line. BBN-AuNP-TDOTA-^67^Ga showed a very high and rapid internalization in cells (almost 25% of the applied radioactivity after 15 min of incubation) with a relatively slow efflux overtime (≈ 15% after 3 h of incubation). The internalization of CBBN-AuNP-TDOTA-^67^Ga was only about 2%, and remained almost constant during 3 h. These results did not translate to the in vivo performance of these ^67^Ga-labeled nanoparticles. In fact, their biodistribution profile in BALB/c nude mice bearing human prostate PC3 xenografts was relatively similar, namely in which concerns the uptake in the organs that overexpress GRP receptors: moderate tumour uptake and low pancreas uptake for both NPs. These results discard, to some extent, that the tumoural uptake mechanism of these nanoparticles in vivo is through an active targeting mediated by GRPr. Eventually, other factors, such as EPR and the protein corona effect, might play prominent role in the in vivo transport of these BBN-containing nanoparticles. Additionally, the administration route also plays an important role on the pharmacokinetic profile of the nanoparticles. After intraperitoneal administration, a lower retention of the radioactive NPs in the RES organs (liver, spleen and lung) is observed, as well as a greater absorption in the pancreas that is accompanied however by a lower tumour uptake. Blocking experiments were done for BBN-AuNP-TDOTA-^67^Ga using the intraperitoneal administration route and after previous treatment of the tumour-bearing mice with free BBN. It was observed a significant decrease (≈34%) of the pancreas uptake but no alteration was observed in the tumour accumulation. These results suggest that the uptake of BBN-AuNP-TDOTA-^67^Ga in the pancreas is possibly mediated by GRPr, while in the case of the tumour uptake, the contribution of the EPR effect seems to be dominant [49].

To further expand the theranostic capabilities of these BBN-AuNP-TDOTA platforms, the authors have also studied their loading with gadolinium aiming to obtain new tools for multimodal SPECT/MRI imaging. Relaxometric studies showed that the Gd-containing AuNPs display contrast properties for MRI T1 and/or T2 relaxometry. Furthermore, radiosensitization studies showed that these AuNPs induce radiotoxic effects in prostate cancer PC3 cells, upon incubation of the cells with the NPs and exposure to a dose of 2 Gy (γ-photons, 1530 keV). These effects were slightly enhanced by the presence of the Gd in the AuNPs. Biodistribution studies were performed for Gd-BBN-AuNP-TDOTA-^67^Ga in PC3-xenograft Balb/c mice after intravenous and intraperitoneal administration of the NPs. The obtained biodistribution pattern is in perfect agreement with that observed for the same AuNPs without Gd. In addition, it was observed a very low uptake in the main organs and a high tumour retention (96.5 ± 26.0% and 76.8 ± 23.3% ID/g at 1 and 24 h after injection, respectively) following the intratumoural administration of the NPs [75].

Niculae et al. have recently evaluated the added value of using gold radionanoplatforms to enhance the intracellular retention of ^68^Ga in tumour cells with respect to the use of the congener radiocomplexes carrying the somatostatin analogs Tyr(3)-octreotide (TOC) and NaI(3)-octreotide (NOC) or a neurotensin (NT) analog. Thus, ^68^Ga-DOTA-TOC, ^68^Ga-DOTA-NOC and ^68^Ga-DOTA-NT were conjugated to AuNPs and evaluated in vitro in human colon cancer cell line (HT-29). ^68^Ga-AuNPDOTA-NOC and ^68^Ga-AuNPDOTA-TOC provide a 35% and 50% improvement relatively to ^68^Ga-DOTA-NOC and ^68^Ga-DOTA-TOC respectively, approximately 40 min after the incubation in HT-29 cells. However, it was found that the gain conferred by ^68^Ga-AuNPDOTA-NT relative to ^68^Ga-DOTA-NT was only approximately 10%, 20 min after incubation in HT-29 cells [76].

#### 4.2.3. Technetium-99m

The emission of favorable low energy γ-rays (140 keV), suitable half-life (6.02 h), easy and economical availability of the ^99^Mo/^99m^Tc generators, justify why ^99m^Tc remains the most widely used SPECT imaging radionuclide in clinics [118]. In the last years, several multifunctional low-generation dendrimer-entrapped gold nanoparticles (DENPs) radiolabeled with ^99m^Tc have been developed and reported in literature. Shen and co-authors were the first to evaluate in vitro and in vivo gold NPs functionalized with low-generation poly(amidoamine) dendrimers (PAMAM) as nanoprobes for dual SPECT/CT imaging. For that purpose, the NPs were modified with folic acid (FA) as a targeting vector and with a DTPA chelator, which were covalently attached to the PAMAM dendrimer. The resulting dendrimer/Au nanoparticles were radiolabeled with ^99m^Tc, showing high colloidal and radiochemical stability and absence of toxicity in HeLa cells, up to concentrations of the order of 400 nM. Studies in HeLa-HFAR cells, that overexpress folic acid receptors, confirmed the specific uptake of the nanoparticles functionalized with folic acid. However, the study of the biodistribution of the ^99m^Tc-labeled dendrimer/AuNPs in a murine HeLa xenograft tumour model showed significantly higher uptake in spleen, lung, liver and kidney than in the tumour [78]. Related ^99m^Tc-dendrimer-nanoplatforms, with acetylated or hydroxylated terminal dendrimers, exhibited good properties for the detection of sentinel lymph node by dual SPECT/CT imaging. On the other hand, it has been shown that ^99m^Tc-dendrimer-AuNPs functionalized with a CXCR4 ligand (FC131 peptide) can specifically target glioma and other types of cancer that overexpress CXCR4 receptors, for use in SPECT/CT dual bioimaging [80]. ^99m^Tc-AuNP-DENPs were also evaluated as SPECT radioprobes for the detection of apoptosis, being proved in vitro that ^99m^Tc AuNP-DENPs decorated with the duramycin peptide have a high propensity for targeted imaging of apoptotic C6 cancer cells. 

^99m^Tc-AuNP-DENPs decorated with a RGD analog peptide showed a favorable profile for targeted SPECT/CT imaging of α_v_β_3_ integrin overexpressing tumours [79,80,81,82]. The capability of pegylated ^99m^Tc-labeled AuNPs decorated with a RGD peptide to effectively target α_v_β_3_ integrin receptors had previously been documented. The preclinical evaluation of these RGD-containing AuNPs showed that the nanoparticles have a high uptake in the lung metastases (14% of the injected dose at 60 min after intravenous injection) of a 4T1 mouse model of breast cancer [83].

Dhawan and co-workers have studied ^99m^Tc-labeled NPs for the non-invasive detection of colon cancer by SPECT imaging. For this purpose, they have conjugated 3,5,4′-trihydroxytrans-stilbene (resveratrol, Res) to the AuNPs in order to increase their selectivity towards colon cancer cells. The accumulation and retention of ^99m^Tc-Res-AuNP in HT 29 colon cancer cells was significantly higher than the congener non-targeted ^99m^Tc-AuNPs. Biodistribution studies performed in rats with colon cancer confirmed that ^99m^Tc-Res-AuNP have an higher uptake ratio colon tumour/normal colon than the non-targeted ^99m^Tc-AuNPs, which leads to an improved tumour to background contrast [84].

Recently, Shi et al. have developed ^99m^Tc-labeled polyethylenimine (PEI)-entrapped AuNPs, functionalized with PEG and alkoxyphenyl acylsulfonamide (APAS) groups (APAS-^99m^Tc-AuPENs). Due to their negatively charged sulfamine groups and positively charged ammonium groups, APAS units are neutral at physiological pH (pH 7.4) and are positively charged at more acidic pH. The authors have considered that this feature could improve the cellular retention of the nanoparticles in cancer cells, which have a mild acid microenvironment. This reasoning was corroborated by the results of in vitro studies performed with the fibrosarcoma HT1080 cell line. It has been observed a higher concentration of radioactivity in the cells treated with APAS-^99m^Tc-AuPENs, at pH 6, when compared with the cells treated with the NPs not functionalized with APAS [85]. Shi and co-workers also developed ^99m^Tc-AuNPs functionalized with Annexin V for in vivo targeting of apoptotic macrophages, which are abundant in atherosclerosis plaques. In vitro studies performed on macrophages (RAW264.7) with apoptosis induction and in vivo studies conducted on high-fat diet fed ApoE^−^/^−^ mice demonstrated the suitability of these nanoparticles to target specifically arteriosclerotic plaques containing apoptotic macrophages [86].

Sakr et al. have investigated ^99m^Tc-labeled AuNPs conjugated with gallic acid and loaded with doxorubicin (^99m^Tc-gallic-AuNPs-DOX) for image-guided drug delivery. The non-labeled AuNPs display suitable in vitro stability in saline and in rat serum for 3 days. Biodistribution studies of the ^99m^Tc-labeled nanoparticles, performed in female albino Swiss mice having Ehrlich ascites carcinoma, showed a considerable tumour uptake of 22.45% ID/g after 2 h of intravenous injection. Furthermore, ^99m^Tc-gallic-AuNPs-DOX displayed a nearly 80% tumour retention upon intratumoural injection, at least for 2 h after administration [87,88,89]. 

Silva et al. have studied AuNPs stabilized with a dithiolated DTPA (DTDTPA), previously developed by Roux and co-workers, as potential glutathione-responsive drug delivery systems. The AuNP-DTDTPA were labeled with the [^99m^Tc(CO)_3_(H_2_O)_3_]^+^ precursor and the resulting radiolabeled NPs were studied in vitro in the presence of glutathione (GSH). The results pinpointed that GSH promotes the cleavage of the disulfide bonds of the polymeric DTDTPA coating, which can be exploited for GSH-mediated delivery of drugs attached at the DTDTPA framework [119,120,121]. 

#### 4.2.4. Indium-111

^111^In is a gamma emitter with a half-life of 2.8 d that is suitable for clinical SPECT imaging. In addition, it also emits Auger electrons that are potentially useful for targeted radionuclide therapy. In the last years, there have been only a few studies reported for AuNPs radiolabelled with ^111^In, seeking to demonstrate their potential interest for imaging and/or therapy [26,122]. These studies include the evaluation of the pharmacokinetics and biodistribution of AuNPs decorated with pMMP9 (pMMP9 = DTPA-Gly-Pro-Leu-Gly-Val-Arg-Gly-Lys-Gly-Tyr-Gly- Ahx-Cys-NH_2_), which is a matrix metalloproteinase-9 (MMP9) cleavable peptide. The pMMP9-containing AuNPs were radiolabeled simultaneously with ^111^In and ^125^I and were evaluated in tumour-bearing mice by in vivo SPECT imaging. At 4 h after intravenous injection, ^111^In was detected mainly in the blood while ^125^I was present in the thyroid, stomach and bladder. This result was attributed to the higher in vivo stability of the ^111^In-radiolabeled moiety if compared with the ^125^I-radiolabeled one. Two types of tumours with different MMP9 expression levels (high = A431; low = 4T1Luc) were implanted in nude mice to explore the ability of the nanoparticles to accumulate in tumours showing MMP9 activity. SPECT/CT images showed that the nanoparticles progressively accumulated in 4T1Luc tumours with low expression of MMP9, reaching 48 h upon intravenous injection a SUV value of 2.8 ± 0.11 (10.2 ± 0.33% ID/g), while a lower SUV of 1.75 ± 0.2 (6.23 ± 0.72% ID/g) was observed in the same period in the A431 tumours with high expression of MMP9. The difference in pharmacokinetics was assigned to the highest MMP9 level in the A431tumours that led to cleavage of the peptide radiolabeled with ^111^In and its clearance from the tumour [90].

AuNPs loaded with the epidermal growth factor (EGF) and radiolabeled with ^111^In (^111^In-EGF-AuNP) were evaluated in vitro using two breast cancer cell lines with different levels of EGFR expression. The ^111^In-labelled EGF-AuNPs presented significantly higher levels of uptake and more pronounced radiotoxicity in MDA-MB-468 cells compared with MCF-7 cells. This reflects the higher EGFR expression (100 times-fold) of MDA-MB-468 cells versus MCF-7 cells [91,123]. In another study, ^111^In-labeled AuNPs decorated with pegylated trastuzumab (trastuzumab-AuNP-^111^In) were evaluated for the targeting of HER2-positive breast cancer cells. Dark field and confocal fluorescence microscopy showed the perinuclear location of trastuzumab-AuNP-^111^In in SK-BR-3 cells having a high HER2 expression. Biodistribution studies of trastuzumab-AuNP-^111^In in mice bearing subcutaneous MDA-MB-361 xenografts have shown a low accumulation of the NPs in the tumour with a high liver uptake [92]. Nevertheless, the intratumoural injection of trastuzumab-AuNP-^111^In, using the same animal model, led to a significant reduction of the tumour mass over 70 days, without apparent toxicity in normal tissues [93].

#### 4.2.5. Gold-198/199 

As can be verified in Table 2, ^198^Au (T_1/2_ = 64.7 h) and ^199^Au (T_1/2_ = 75.3 h) are relatively long-lived β^−^ emitters that are suitable for therapeutic use. In addition, both radionuclides emit also γ-photons that allow SPECT imaging studies. In the case of ^199^Au, 5 nm gold nanoparticles doped with ^199^Au decorated with D-Ala1-peptide (DAPTA) have been evaluated for in vivo target of the C-C chemokine receptor 5 (CCR5), overexpressed in triple negative breast cancer (TNBC). NanoSPECT/CT images obtained 24 h after intravenous injection of ^199^AuNP-DAPTA in a 4T1 TNBC orthotopic mouse model showed a heterogeneous pattern of penetration and retention within the tumour, in addition to high liver and spleen accumulation. The images are in full agreement with the results of biodistribution studies, which showed a tumour uptake of 7.13 ± 0.08% ID/g and a ratio tumour/muscle of 18.7 ± 1.69. All together these results led the authors to conclude that ^199^AuNP-DAPTA is a promising nanoplatform for the CCR5-targeted imaging of triple breast cancer [100].

Biodistribution studies of ^199^Au-labeled AuNPs decorated with a non-specific antibody (Bharglob) in normal rats showed that the accumulation of radioactivity occurs predominantly in stomach and organs of the RES system, at 24 h after injection. In an attempt to minimize the unfavorable pharmacokinetics observed, non-specific gammaglobulin was co-administered and a considerable decrease in the RES uptake was observed (about 50%) [101]. 

Loyalka and co-authors estimated the dose distribution delivered by ^198/199^Au-labeled AuNPs to the tumour sites, inside the human prostate, as well as to the surrounding normal tissues using the Monte-Carlo N-Particle code (MCNP-6.1.1 code). A simple geometric model of the tumour, prostate, bladder and rectum was constructed. MCNP simulations showed that the doses are deposited homogenously and mostly within the tumour and marginally in the bladder and rectum. However, the dose deposited by ^198^Au is significantly higher than the dose deposited by ^199^Au in the tumour region, as well as in normal tissues [102].

Katti et al. have reported the synthesis of radioactive ^198^Au-AuNPs functionalized with mangiferin (MGF) [56]. The specificity of MGF towards the laminin receptor promoted the accumulation of the AuNPs in prostate tumours (PC-3) induced in mice. Detailed in vivo therapeutic efficacy studies, through the intratumoural delivery of the AuNPs, showed retention of over 80% of the injected dose in tumours up to 24 h. By three weeks post treatment, tumour volumes of the treated group of animals showed an over 5 fold reduction as compared to the control saline group.

Chakravarty et al. have developed ^198^Au-AuNPs functionalized with a RGD peptide derivative and studied their suitability for melanoma cell targeting [124]. In vitro studies showed that the AuNPs bind to murine melanoma B16F10 cells with high affinity and specificity. Biodistribution studies of the AuNPs administered intravenously in melanoma tumour bearing C57BL/6 mice showed high uptake in the tumour within 4 h post-injection, with significant decrease at the same time point when co-injected with a blocking dose of the RGD peptide. Radiotherapy studies in melanoma tumour bearing mice showed significant regression of tumour growth without apparent body weight loss over the course of 15 days.

## 5. Examples of Radiolabeled AuNPs for Therapeutic Applications

Besides ^198^Au and ^199^Au, various other therapeutic radionuclides of the β^−^ or α-emitting types were used to label AuNPs aiming to obtain enhanced therapeutic effects, namely within a theranostic approach of cancer. As resumed in Table 4 and reviewed below, part of these studies comprised also SPECT imaging experiments since some of these radionuclides also emit γ photons during their decay and, for this reason, are also suitable for in vivo imaging.

### 5.1. Beta-Emitting Isotopes

#### 5.1.1. Yttrium-90

Yttrium-90 (^90^Y) is a β^−^ emitter decaying to ^90^Zr with a half-life of 64.6 h and with a decay energy of 2.28 MeV. It is a hard β^−^ emitter and the emitted particles can penetrate tumour soft tissue to a length of 11 mm. For this reason, ^90^Y leads to important cross-fire effects and does not require its accumulation in every tumour cell to produce deleterious radiotoxic effects. However, it can kill non-targeted cells in the vicinity of the target tumours. Ghandehari et al. have reported on the use of AuNRs to increase hyperthermia in tumours and to enhance the radiotherapeutic effect of a ^90^Y-labeled N-(2-hydroxypropyl) methacrylamide (HPMA) copolymer [126]. The macromolecular nature of HPMA allows it to passively target tumours through the EPR effect. Prostate tumour animal models were treated with a co-injection of PEGylated AuNRs and ^90^Y-labeled HPMA, and thereafter were submitted to laser treatment to induce localized hyperthermia. Results showed an increase in the uptake of radiolabeled copolymer in the hyperthermia treated prostate tumours, with no significant accumulation in non-targeted tissues. Additionally, the highest reduction in tumour growth was observed in the tumours submitted to hyperthermia and treated with ^90^Y-labeled HPMA copolymer conjugates. Althoughmost radioactivity accumulation was found in the tumours, the biodistribution studies also showed a significant uptake in the kidneys; however, the histological studies did not show any pronounced damage in the primary organs of the mice. 

Reilly et al. have performed in vivo imaging and Monte Carlo simulations of nanoparticle depots (NPD) [134]. consisting of a porous calcium alginate platform loaded with AuNPs coated with PEG and polyglutamide, and functionalized with a DOTA derivative for radiolabeling with ^111^In, ^90^Y and ^177^Lu [125]. The studies were performed in a way to compare these NPDs with conventional permanent seed implantation (PSI) brachytherapy in mice bearing subcutaneous human breast cancer xenografts. For the simulated NPDs, ^90^Y delivered the most homogeneous dose distribution.

#### 5.1.2. Iodine-131

Iodine-131 (^131^I) has a half-life of 8.02 d and emits β^−^ particles and γ radiation. It decays in two steps to form the stable 1^31^Xe, initially through beta decay (606 keV) followed rapidly after by gamma emission (364 keV). Lan et al. have reported on the synthesis of ^131^I-labelled AuNRs decorated with a cyclic RGD peptide derivative for integrin *α*_v_*β*_3_ receptor targeting, which is responsible for tumour angiogenesis [59]. Results showed that the AuNPs were selectively taken up by the tumour in murine melanoma B16F10 cancer bearing mice mainly via integrin *α*_v_*β*_3_-receptor mediated endocytosis, after intravenous administration. However, the biodistribution studies also showed higher uptakes in organs of the RES, such as liver, spleen and lungs, most likely due the large size of the AuNRs (93.4 nm, lenght). Additionally, when administered in breast cancer MCF7 tumour bearing mice, there was no significant uptake in the tumours, which was attributed to the low *α*_v_*β*_3_ receptor expression in this cell line in comparison with B16F10.

Zhao et al. have studied polyethylenimine-entrapped AuNPs functionalized with a chlorotoxin (CTX) peptide and labeled with ^131^I for SPECT/CT imaging and radionuclide therapy of glioma [127]. CTX is a peptide capable of targeting various cancer cells including glioma, sarcoma and prostate, and capable of permeating the blood brain barrier (BBB) intact. The AuNPs were entrapped inside a polyethylenimine polymeric nanoparticle, functionalized with PEG, chlorotoxin, and 3-(4-hydroxyphenyl)propionic acid-OSu (HPAO). The presence of the HPAO allowed for a facile radiolabeling with ^131^I. After intravenous injection of the radiolabeled NPs in a subcutaneous glioma-bearing mice, it was possible to visualize through SPECT imaging a high tumour accumulation, with the highest uptake at 8 h p.i. The congener NPs, without the CTX, still displayed some significant tumour uptake at 8 h p.i., but it was less than half intensity when compared with the ones bearing peptide. These results also translated to the in vivo studies performed in orthotopic rat glioma models, where the CTX-containing NPs displayed a significant SPECT signal, which peaked at 8 h p.i., demonstrating the capability of these NPs to cross the BBB. 

#### 5.1.3. Lutetium-177

Lutetium-177 (^177^Lu) has a half-life of 6.7 d and undergoes β^−^ decay, emitting β^−^ particles (134 keV) but also γ radiation (208 keV). As mentioned in the introduction, ^177^Lu is a soft β^−^ emitter with increasing clinical impact on PRRT of cancer. The emission of γ photons allows for the use of ^177^Lu in preclinical SPECT imaging.

Ferro-Flores et al. have developed a DOTA-dendrimer-folate-bombesin conjugate that was used to entrap AuNPs in the dendritic cavity (DenAuNP-folate-bombesin). The presence of the folate and bombesin was to improve affinity of the NPs to the folate receptor and gastrin releasing peptide receptor, respectively, which are overexpressed in certain types of breast cancer cells. The entrapped AuNPs provided photophysical properties to the whole nanoconjugate suitable for optical imaging. The final nanoconjugate was labeled with ^177^Lu, seeking for multimodal platforms suitable for breast cancer cell targeting [128,129]. The radiolabeled nanoconjugate showed specific uptake in breast cancer T47D cells and provided suitable optical images. Plasmonic–photothermal therapy studies in T47D cells incubated with DenAuNP-folate-bombesin showed a higher increase in medium temperature (46.8 °C), compared with the congeners without the entrapped AuNPs (39.1 °C), which consequently led to a more significant decrease in cell viability [132]. Moreover, preliminary in vivo studies showed quantitative tumour retention 96 h after intratumoural administration of the ^177^Lu-labeled DenAuNP-folate-bombesin in breast cancer T47D tumour bearing mice [128]. 

The group of Reilly et al. has studied the in vivo stability of AuNPs functionalized with different PEG derivatives containing DOTA for ^177^Lu labeling [131]. These PEG derivatives varied on their thiol group responsible for AuNP surface conjugation, including monothiol, dithiol and multithiol groups. Biological studies showed that the AuNPs containing the multi-thiol PEGs displayed the highest stability in vitro and the lowest liver uptake in vivo. The group also developed new AuNPs constructs functionalized with monothiolated PEG chains linked to DOTA and with panitumumab for epidermal growth factor receptor (EGFR) targeting [130]. These multifunctional AuNPs were labeled with ^177^Lu and underwent a preclinical study as nanoseeds for brachytherapy of locally advanced breast cancer. 

The preclinical studies of the panitumumab-containing AuNPs comprised the intratumoural administration of the ^177^Lu-labeled nanoconstructs in CD-1 athymicmice bearing subcutaneous MDA-MB-468 xenografts. It was observed a high radioactivity concentration in the tumour at 1 h post-injection, but with a significant decrease (2–3 fold) after 48 h of administration. However, when compared with the respective congeners without the EGFR-targeting panitumumab, the uptake in tumour was not significantly different; and in both cases, some accumulation in non-targeted organs like liver and spleen had increased about 3–4 fold between 1 to 48 h p.i. Dosimetry studies estimated that the tumour receives the highest dose, and the liver, spleen and pancreas are the non-targeted organs more exposed to radioactivity. Long-term treatment studies showed the inhibition of tumour growth in mice treated with both targeted and non-targeted ^177^Lu-labelled AuNPs, without toxicity in normal tissues. Additionally, non-labelled NPs did not display any visible tumour growth inhibition [130].

### 5.2. Alpha-Emitting Isotopes

#### 5.2.1. Astatine-211

Astatine-211 (^211^At) has a half-life of 7.21 h and undergoes a branched decay: 41.8% decays to ^207^Bi (T_1/2_ = 32.9 y) with α emission (5.9 MeV); 58.2% decays by electron capture to ^211^Po (T_1/2_ = 516 ms) that then quickly decays to the stable ^207^Pb with α emission (7.5 MeV). Majkowska-Pilip et al. have reported on AuNPs modified with PEG chains and attached to the antibody trastuzumab. This antibody not only possesses chemotherapeutic properties but it also has affinity towards HER2 receptors, which are overexpressed in certain breast cancer cells [132]. The AuNPs were labeled with ^211^At by adsorption of the radionuclide to the nanoparticle surface by taking advantage of the high affinity of gold for heavy halogens. In vitro biological studies showed a higher affinity and cytotoxicity for the trastuzumab-containing AuNPs, compared with the ones without the antibody, towards HER2-overexpressing human ovarian SKOV-3 cells. Additionally, it was also verified that the trastuzumab-containing AuNPs were able to internalize into the cells and deposited near the nucleus. 

#### 5.2.2. Actinium-225

Actinium-225 (^225^Ac) is a radionuclide that decays to ^221^Fr with a T_1/2_ of 10.0 d and through α emission (5.94 MeV). Bouziotis et al. have reported on the synthesis of AuNPs functionalized with a thioctic acid-modified DOTAGA and radiolabeled with ^225^Ac. The resulting ^225^Ac-labeled AuNPs were evaluated as an injectable radiopharmaceutical form of brachytherapy for local radiation cancer treatment, using cellular and animal models of glioblastoma multiforme [133]. In vitro radiocytotoxicity studies in glioblastoma multiforme U87MG cells showed a significant cell death upon exposure to the radiolabeled AuNPs. Consistently, their intratumoural administration in U87MG tumour bearing mice resulted in the retardation of tumour growth, even with a low injected dose (1 kBq) per mice. However, while biodistribution studies showed the highest uptake of radioactivity in the tumours, some significant accumulation in non-targeted organs like liver, kidneys and spleen was observed. 

### 5.3. Boron Neutron Capture Therapy (BNCT)

BNCT is a technique based on the nuclear reaction ^10^B(n, α)^7^Li that involves a neutron-capture process causing fission reactions that originate high-LET alpha particles (150 keV/μm) [135]. BNCT differs from the classical radionuclide therapy discussed previously, as the therapeutic radiation delivered by BNCT is triggered by the external neutron irradiation of the boron atoms that are accumulated by tumour cells. However, both methodologies are categorized as internal radionuclide therapies, and BNCT has been gaining increased interest in the last few years, with some examples reported in literature combining AuNPs with this technique.

Llop et al. recently synthesized AuNPs (core diameter of 19.2 ± 1.4 nm; hydrodynamic diameter of 37.8 ± 0.5 nm) functionalized with PEG and an anion-rich cobalt bis (dicarbollide), commonly known as COSAN, and evaluated its potential for BNCT in mouse model xenografts with human fibrosarcoma HT1080 cells [136]. These COSAN-containing NPs have been radiolabelled with ^124^I (in two different positions, the core and the shell) to allow tracking of their biodistribution pattern by PET imaging. The studies showed that the radiolabeled nanoparticles are stable in vivo, since no significant iodine accumulation was detected in the thyroid during the imaging studies (0–144 h). However, no significant uptake was found in the tumours. The maximum values obtained are below 0.5% ID cm^−3^, regardless of the labelling approach used (core vs shell labelling), and progressively decreased over time being almost undetectable at t > 72 h.

To improve the tumour uptake and retention of boron cage-containing AuNPs for BNCT, the same authors synthetized smaller gold nanoparticles (core size = 4.1 ± 1.5 nm, hydrodynamic diameter of 39.6 ± 0.8 nm) loaded with PEG, COSAN and functionalized with tetrazine (Tz) units [137]. To enable the in vivo screening of the biodistribution of AuNPs by positron emission tomography (PET), the nanoparticles were radiolabelled with [^64^Cu] CuCl_2_ (core labelling). PET biodistribution studies were conducted in cancer-xenograft bearing mouse model with HER2 positive BT-474 breast cancer cells, using both a simple and a pre-targeting strategy. For a pre-targeting approach, Trastuzumab, an antibody that selectively binds to HER2, was functionalized with trans-cyclooctene (TCO) ligand, to promote the in vivo click reaction with tetrazine present in the radiolabeled AuNPs and was administered intravenously 24 h before the AuNPs. PET-CT scans were performed ≈ 1, 6, 24, and 48 h. The accumulation in the tumours was clearly visualized by PET images. No added value was observed from the pre-targeting approach. Indeed, the maximum tumoural uptake was achieved at t = 24 h after AuNP injection, with values of 4.76 ± 1.85% ID cm^−3^ and 4.42 ± 2.08% ID cm^−3^ for pre-targeting and normal strategy, respectively.

## 6. Concluding Remarks

In this review, we have highlighted some of the most relevant AuNPs developed in the last few years for nuclear medicine applications, particularly for cancer treatment. To this date, AuNPs have provided interesting platforms for the delivery of radioisotopes to cancer cells and tissues, with the advantage of their low toxicity, biocompatibility and enhanced biological half-life. Despite these encouraging progresses, there is the need to optimize the efficacy of most radiolabeled AuNPs to obtain new nanotools with clinical usefulness. To fulfil this goal, still is necessary the development of novel AuNPs core structures and their controlled modification with different molecular entities to further improve their pharmacological profile. In this respect, one can profit from recent achievements on site-specific approaches to modify clinically relevant biomolecules and from technological advances in the production of innovative medical radionuclides. 

Having in consideration the reported preclinical studies, radiolabeled AuNPs are not expected to be a valuable alternative to more conventional molecular radio-pharmaceuticals designed for systemic administration, such as radiolabeled peptides or antibodies that are already in clinical use for peptide receptor radionuclide therapy (PRRT) or radioimmunotherapy (RIT). In fact, a large majority of the tested radiolabeled AuNPs have shown a sub-optimal biodistribution with more or less prolonged accumulation in non-target organs, mainly the liver and spleen, which can lead to unfavorable radiation dosimetry. By contrast, we consider that the use of radiolabeled AuNPs in combination with topical administration, as a kind of “nanoseeds”, might open new avenues in cancer theranostics with minimization of detrimental side effects. This is particularly true when using therapeutic radionuclides that emit high LET and short-range particle radiation, as is the case of alpha or Auger emitters. In this respect, AuNPs are clearly advantageous over classical molecular radiopharmaceuticals. The NPs are expected to exhibit a higher retention in the tumors, following their intratumoral administration, when having the proper size, shape, charge and/or coating. Moreover, the development of multifunctional “nanoseeds” for the simultaneous delivery of radionuclides, cytotoxic drugs and/or radiosensitizers will allow combined chemo- and radiotherapy regimens with a better chance to surpass radio/chemoresistance processes. Hence, it is our conviction that AuNPs can play a role in future applications of nuclear medicine by providing unique combinations of imaging and therapy modalities to improve the diagnosis, treatment and management of cancer.

## Figures and Tables

**Table 1:** Examples of Relevant Radionuclides for Imaging Use.

Radionuclide	Half-Life	Mode of Decay (%)	Application
^11^C	20.3 min	β^+^ (100)	PET
^18^F	109.8 min	β^+^ (97) EC ^a^ (3)	PET
^61^Cu	3.3 h	β^+^ (100)	PET
^62^Cu	9.7 min	β^+^ (100)	PET
^64^Cu	12.7 h	β^−^ (40) β^+^ (19) EC (41)	PET/Therapy
^67^Ga	3.27 d	EC (100)	SPECT
^68^Ga	67.8 min	β^+^ (90) EC (10)	PET
^86^Y	14.7 h	β^+^ (33) EC (66)	PET
^89^Zr	78.4 h	β^+^ (100)	PET
^99m^Tc	6.0 h	IT ^b^ (100)	SPECT
^111^In	2.83 d	EC (100)	SPECT
^123^I	13.2 h	EC (100)	SPECT
^124^I	100.8 h	β^+^ (100)	PET

^a^ EC = Electron Capture; ^b^ IT = Isomeric Transition.

**Table 2:** Examples of Relevant Radionuclides for Therapeutic Use.

Radionuclide	Half-Life (h)	Mode of Decay (%)
^67^Cu	61.8	β^−^ (100)
^90^Y	64.1	β^−^ (100)
^131^I	192.0	β^−^ (100)
^153^Sm	46.3	β^−^ (100)
^166^Ho	26.8	β^−^ (100)
^177^Lu	161.0	β^−^ (100)
^186^Re	89.2	β^−^ (92) EC ^a^ (8)
^188^Re	17.0	β^−^ (100)
^198^Au	64.7	β^−^ (100)
^199^Au	75.3	β^−^ (100)
^211^At	7.2	α (100)
^223^Ra	274.5	α (100)
^225^Ac	238.1	α (100)

^a^ EC = Electron Capture.

**Table 3:** Examples of AuNPs labeled with imaging radionuclides and respective preclinical studies.

Radioisotope	Type of AuNPs/Size/Coating (Radiolabeling Approach)	Imaging Application/Study	References
^18^F	Spherical AuNPs/12 nm/LPFFD (^18^F-fluorobenzoate conjugation)	Biodistribution studies and in vivo PET imaging in healthy mice.	[63,64,65]
^64^Cu	AuNSs/120 nm/cyclic-RGD (Chelator-based)	PET imaging and thermoablation treatment in HCT116 human colorectal cancer xenograft mice.	[66]
	Spherical AuNPs/9.4 nm/PEG (^64^Cu/Au alloying)	Biodistribution and in vivo PET imaging in rats bearing EMT-6 breast cancer.	[61]
	AuNSs/44.7 nm/doxorubicin, lipiodol (chelator-based)	Biodistribution and chemotherapeutic drug delivery studies, laser induced thermal treatment and in vivo PET imaging in hepatic VX2 tumours in mice.	[67,68]
	AuNCs/35 nm/PEG, α-MSH (chelator-based)	Biodistribution studies and PET/CT imaging in vivo in B16/F10 melanoma mouse model.	[69]
	Spherical AuNPs/3 nm/PEG, bombesin, LUG, NIR dye SIDAG (chelator-based)	In vitro radiotoxicity studies in PC3 and LNCaP cell lines. Biodistribution studies and PET/CT imaging in healthy mice.	[70]
	Nanoclusters/4.2 nm/AMD3100 (chelator-based)	Biodistribution studies and PET/CT imaging in 4T1 mouse orthotopic breast cancer mouse model with lung metastases.	[71]
	Spherical, hexapodal and rod shaped AuNPs/10, 30, 80 nm/PEG, cyclic RGD (^64^Cu epitaxial growth on NP surface)	Biodistribution studies and in vivo PET imaging in U87MG glioblastoma xenograft mice.	[62]
	Tripod shaped AuNPs/25, 35 nm/DAPTA (^64^Cu-doped NPs)	In vivo PET imaging and image-guided photothermal treatment in 4T1-TNBC xenograft mice.	[72]
	AuNCs/30.4, 54.4 nm/PEG (chelator-based)	Biodistribution and in vivo PET imaging in EMT-6 murine breast cancer mouse model.	[73]
	AuNSs/120 nm/PEG (chelator-based)	In vivo PET imaging of ^64^Cu-NS-RGDfKS pharmacodynamics in nude rats xenografted with head and neck squamous cell carcinoma (HNSCC)	[74]
^67^Ga	Spherical AuNPs/4 nm/bombesin, DOTA (chelator-based)	In vitro radiotoxicity studies in PC3 cells. Biodistribution studies in PC3 xenograft mice.	[49,75]
^68^Ga	Spherical AuNPs/42.5 nm/NOC, TOC (chelator-based)	In vitro binding kinetics studies in human colon cancer cell line (HT-29) and AR42J cell line of acinar pancreatic rat.	[76]
	Spherical AuNPs/3 nm/PEG, bombesin, LUG, NIR dye SIDAG (chelator-based)	Ex vivo biodistribution studies and in vivo fluorescence imaging in LNCaP tumour bearing mice.	[77]
^99m^Tc	Dendrimer-entraped spherical AuNPs/1.6 nm (Au core), 291.2 nm (dendrimer)/PAMAM (chelator-based)	Biodistribution studies in xenograft mice tumours with HeLa cells.	[78]
	Dendrimer-entraped spherical AuNPs/2–6 nm (Au core), 127–139 nm (dendrimer)/PEG, cyclic RGD (chelator-based)	Ex vivo biodistribution studies in albino mice. In vivo Micro-SPECT/CT imaging, in albino mice and nude mice bearing C6 xenografted tumours. Therapeutic efficacy studies in C6 xenografted mice.	[79,80,81,82]
	Spherical AuNPs/5 nm/cyclic RGD (chelator-based)	Scintigraphy imaging in xenografted mice harboring 4T1 metastasis breast cancer.	[83]
	Spherical AuNPs/16.7 nm/Resveratrol (chelator-based)	In vivo biodistribution studies in HT 29 tumour bearing rats.	[84]
	PEI-entraped spherical AuNPs/3.3 nm (Au core)/PEG, fluorescein isothiocyanate, alkoxyphenyl acylsulfonamide (chelator-based)	In vitro CT and SPECT imaging of fribrosarcoma HT1080 cells.	[85]
	Spherical AuNPs/30.2 nm/Annexin V (chelator-based)	SPECT/CT imaging of mice with high fat diet-induced atherosclerosis.	[86]
	Spherical AuNPs/58.9 nm/doxorubicin, EGCG (chelator-based)	In vitro cytotoxicity studies in breast carcinoma MCF-7 and hepatocellular carcinoma HepG-2 cell lines. Biodistribution studies in Ehrlich ascites carcinoma tumour bearing albino mice.	[87]
	Spherical AuNPs/10 nm/gallic, doxorubicin (chelator-based)	In vitro anti-proliferative activity studies in MCF7 cell lines. Biodistribution studies in Ehrlich ascites carcinoma tumour bearing albino mice.	[88,89]
^111^In	Spherical AuNPs/10 nm/MMP9 (chelator-based)	In vivo SPECT/CT imaging in nude mice bearing bilateral tumours (A431 with high MMP9 expression and 4T1Luc with low MMP9 expression).	[90]
	Spherical AuNPs/14 nm/EGF (chelator-based)	Internalization and radiotoxicity studies in MDA-MB-468 and MCF-7 cells.	[91]
	Spherical AuNPs/30 nm/trastuzumab (chelator-based)	Micro-SPECT/CT imaging in MDA-MB-361 human breast cancer xenograft mice.	[92,93]
^124^I	Spherical AuNPs/5, 10, 20 nm/oligotyrosine (^124^I-embeded NPs)	Dendritic cell and macrophages labeling in vivo for PET imaging detection of Sentinel Lymph Nodes.	[94,95,96]
	Crushed Au Shell-covered spherical AuNPs/0.25 nm/ poly(N-vinyl-2-pyrrolidone (chloramine T oxidation combined with ^124^I-embeded NPs)	PET/CT imaging in 4T1 and CT26 tumour bearing mice and photothermal therapy in CT26 tumour bearing mice.	[97,98]
^125^I	Spherical and rod shaped AuNPs/56 nm/cyclic RGD (NP adsorption)	Biodistribution studies and SPECT/CT imaging in H1299 tumour bearing mice.	[99]
	Spherical AuNPs/10 nm/MMP9 (chelator-based)	In vivo SPECT/CT imaging in nude mice bearing bilateral tumours (A431 with high MMP9 expression and 4T1Luc with low MMP9 expression).	[90]
	Spherical AuNPs/5, 10, 20 nm/ ogotyrosine (chloramine T oxidation combined with ^125^I-embeded NPs)	Dendritic cell and macrophages labeling in vivo for SPECT/PET imaging detection of Sentinel Lymph Nodes.	[94,95,96]
^199^Au	Spherical AuNPs/5, 18 nm/DAPTA (^199^Au-doped NPs)	Biodistribution studies and SPECt/CT imaging in 4T1 tumour bearing mice.	[100]
	Amorphous/25–85 nm/PEG, folic acid, human immunoglobulin, Bharglob, M3-monoclonal antibody (^199^Au NP synthesis)	In vivo biodsitribution studies in healthy mice.	[101]
	Spherical AuNPs/not applicable (^199^AuNPs)	Assessment of dose distribution in human prostate cancer using Monte-Carlo simulations.	[102]

**Table 4:** Examples of AuNPs labeled with therapeutic radionuclides and respective preclinical studies.

Radioisotope	Type of AuNPs/Size/Coating (Radiolabeling Approach)	Application/Study	Refs.
^90^Y	Spherical AuNPs-loaded nanoparticle depots/15 nm/PEG, polyglutamide (chelator-based)	Monte Carlo simulations of permanent seed implantation brachytherapy.	[125]
	AuNRs/40 nm/PEG (chelator-based)	Biodistribution studies, combined radiotherapy and hyperthermia treatment in prostate DU145 xenograft mice.	[126]
^131^I	PEI-entraped spherical AuNPs/4.4 nm (AuNP core), 151 nm (PEI)/HPAO, PEG, CTX (chloramine T oxidation)	Targeted SPECT/CT imaging and radionuclide therapy in subcutaneous glioma tumour model in vivo.	[127]
	AuNRs/93 nm/PEG, cyclic RGD (NP adsorption)	SPECT/CT imaging and biodistribution analyses in B16F10 and MCF7 tumour bearing mice	[59]
^177^Lu	Dendrimer-entraped spherical AuNPs/2.5 nm (AuNP core), 5.6 nm (dendrimer)/folate, bombesin (chelator-based)	Radiocytotocixity studies in T47D cells. Biodistribution studies and optical imaging in T47D xenograft mice.	[128,129]
	Spherical AuNPs/30 nm/orthopyridyl disulfide, PEG, panitumumab(chelator-based)	Biodistribution/radiotoxicity studies and small-animal SPECT/CT imaging in MDA-MB-468 xenograft mice.	[130,131]
	AuNRs/15 nm/PEG, polyglutamide (chelator-based)	Monte Carlo simulations of permanent seed implantation brachytherapy.	[125]
^198^Au	Spherical AuNPs/12.5 nm/cyclic RGD (^198^Au NP synthesis)	Biodistribution and tumour regression studies in melanoma C57BL/6 tumour bearing mice.	[124]
	Spherical AuNPs/35 nm/mangiferin (^198^Au NP synthesis)	Biodistribution and therapeutic efficacy studies in prostate PC3 xenograft mice.	[56]
^211^At	Spherical AuNPs/5 nm/PEG, trastuzumab (NP adsorption)	In vitro radiotoxicity studies in human ovarian cancer cell line SKOV-3	[132]
^225^Ac	Spherical AuNPs/2–3 nm/DOTAGA (chelator-based)	Biodistribution and therapeutic efficacy studies in glioblastoma multiform cell line U87 xenograft mice.	[133]

## Abbreviations

**α**	Alpha
α-MSH	α-Melanocyte-stimulating hormone
AuNCs	Gold nanocages
AuNP	Gold nanoparticle
AuNR	Gold nanorod
AuNS	Gold nanoshell
APTES	3-Aminopropyltriethoxysilane
β	Beta
BBB	Blood brain barrier
BBN	Bombesin
BNCT	Boron neutron capture therapy
CLI	Cerenkov luminescence imaging
CT	Computed tomography
CTAB	Cetyltrimethylammonium bromide
CTX	Chlorotoxin
DAPTA	D-Ala1-peptide T-amide
DC	Dendritic cell
DLS	Dynamic light scattering
DOTA	1,4,7,10-Tetraazacyclododecane-1,4,7,10-tetraacetic acid
DOX	Doxorubicin
DPLNs	Draining popliteal lymph nodes
DTDTPA	Dithiolated DTPA
DTPA	Diethylene triamine pentaacetic acid
EC	Electron capture
ECIS.	European Cancer Information System
EGFr	Epidermal growth factor receptor
EPR	Enhanced permeability and retention
GRPr	Gastrin releasing peptide receptor
HNSCC	Head and neck squamous cell carcinoma
HPAO	3-(4-Hydroxyphenyl)propionic acid-OSu
HPMA	N-(2-Hydroxypropyl) methacrylamide
HER	Human epidermal growth factor receptor
IT	Isomeric transition
LET	Linear energy transfer
LPFFD	Cys-Leu-Pro-Phe-Phe-Asp
MC1R	Melanocortin 1 receptor
MRI	Magnetic resonance imaging
NAA	Neutron activation analysis
NET	Neuroendocrine tumours
NIR	Near-infrared
NMR	Nuclear magnetic resonance
NODAGA	1,4,7-Triazacyclononane-1-glutaric acid-4,7-acetic acid
NOTA	1,4,7-Triazacyclononane-1,4,7-triacetic acid
NP	Nanoparticle
NPD	Nanoparticle depots
NTA	Nanoparticle tracking analyses
PEG	Polyethylene glycol
PET	Positron emission tomography
p.i	Post-injection
PRRT	Peptide receptor radionuclide therapy
PSI	Permanent seed implantation
PSMA	Prostate-specific membrane antigen
RES	Reticuloendothelial system
RGD	Cyclic arginine-glycine-aspartic acid
RIe-AuNP	Radionuclide-embedded gold nanoparticles
ROI	Region of interest
SEM	Scanning electron microscopy
SFB	N-Succinimidyl-4-fluorobenzoate
SPECT	Single-photon emission cumputed tomography
SPR	Surface plasmon resonance
SUV	Standardized uptake value
TATE	Octreotate
TEM	Transmission electron microscopy
TNBC	Triple-negative breast cancer
TNF-α	Tumour necrosis factor alpha
XPS	X-ray photoelectron spectroscopy
XRD	X-ray diffraction
